# Effect of Exchange Through Kendo on the Image of the elderly of Junior High School Kendo Club Members

**DOI:** 10.1093/geroni/igab046.2828

**Published:** 2021-12-17

**Authors:** Daisuke Matsumoto, Hayato Uchida

**Affiliations:** University of Hyogo, Himeji-city, Hyogo, Japan

## Abstract

This study targeted junior high school kendo club members who have practice experience with elderly kendo practitioners and examined the image of the elderly and the related factors that intergenerational exchange through kendo brings to the junior high school members. Kendo is one of the traditional Japanese martial arts. The subjects in this research were 193 players who practiced with the elderly kendo practitioners in Osaka prefecture. As a result of factor analysis to clarify the structure of the image, the "evaluation" factor and the "activity / competence" factor were extracted as in the previous research, and it was suggested that the junior high school players generally had positive image regarding the elderly practitioners. As a result of logistic regression analysis to clarify the factors related to the high/ low scores in the image of elderly kendo practitioners, "evaluation" factor showed a significant relationship between "intimacy with elderly practitioners" and "empathic interest", and "activity / competence" factor indicated a significant relationship between "gender" and "intimacy with elderly kendo practitioners." Although it is pointed out that the traditional style of kendo and the image of elderly kendo practitioners have a negative impact on children, it may be possible to control these factors through an appropriate guidance and approach depending on the children's stage of growth .

